# Supplementary figures and images for: Conserved chromatin regulators control the transcriptional immune response to intracellular pathogens in Caenorhabditis elegans
Source: PLoS Genet. 2025 Apr 7;21(4):e1011444. doi: 10.1371/journal.pgen.1011444 (PMC11975079; doi:10.1371/journal.pgen.1011444)

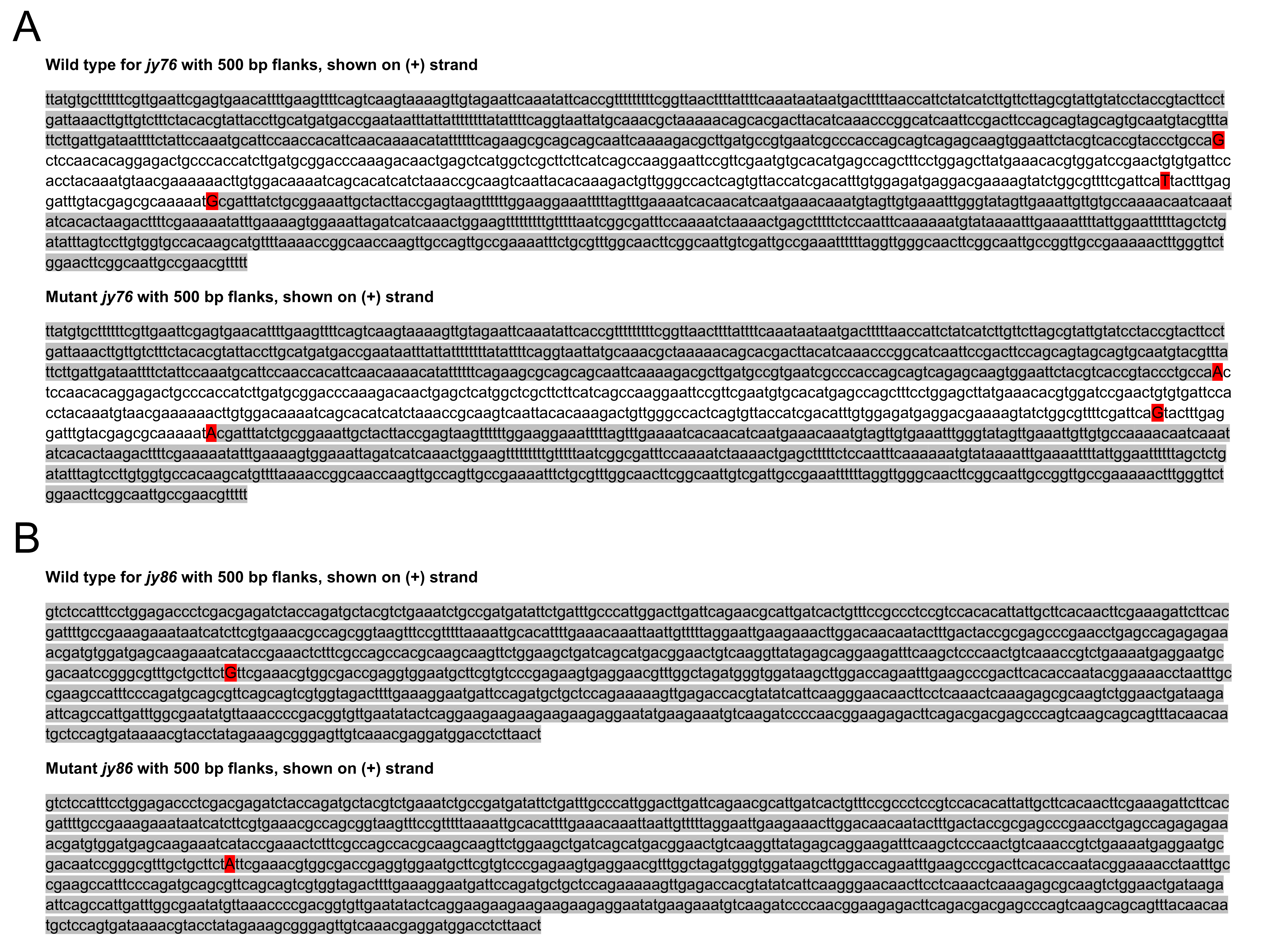

Supplement: S1 Fig — (A, B) Wild-type and mutated nucleotides in jy76 (A) and jy86 (B) are shown on the positive strand, with mutated nucleotides highlighted in red boxes (uppercase letters).Surrounding sequences, including 500 nucleotides upstream and downstream, are displayed in gray boxes (lowercase letters). (TIF) [file pgen.1011444.s001.tif]

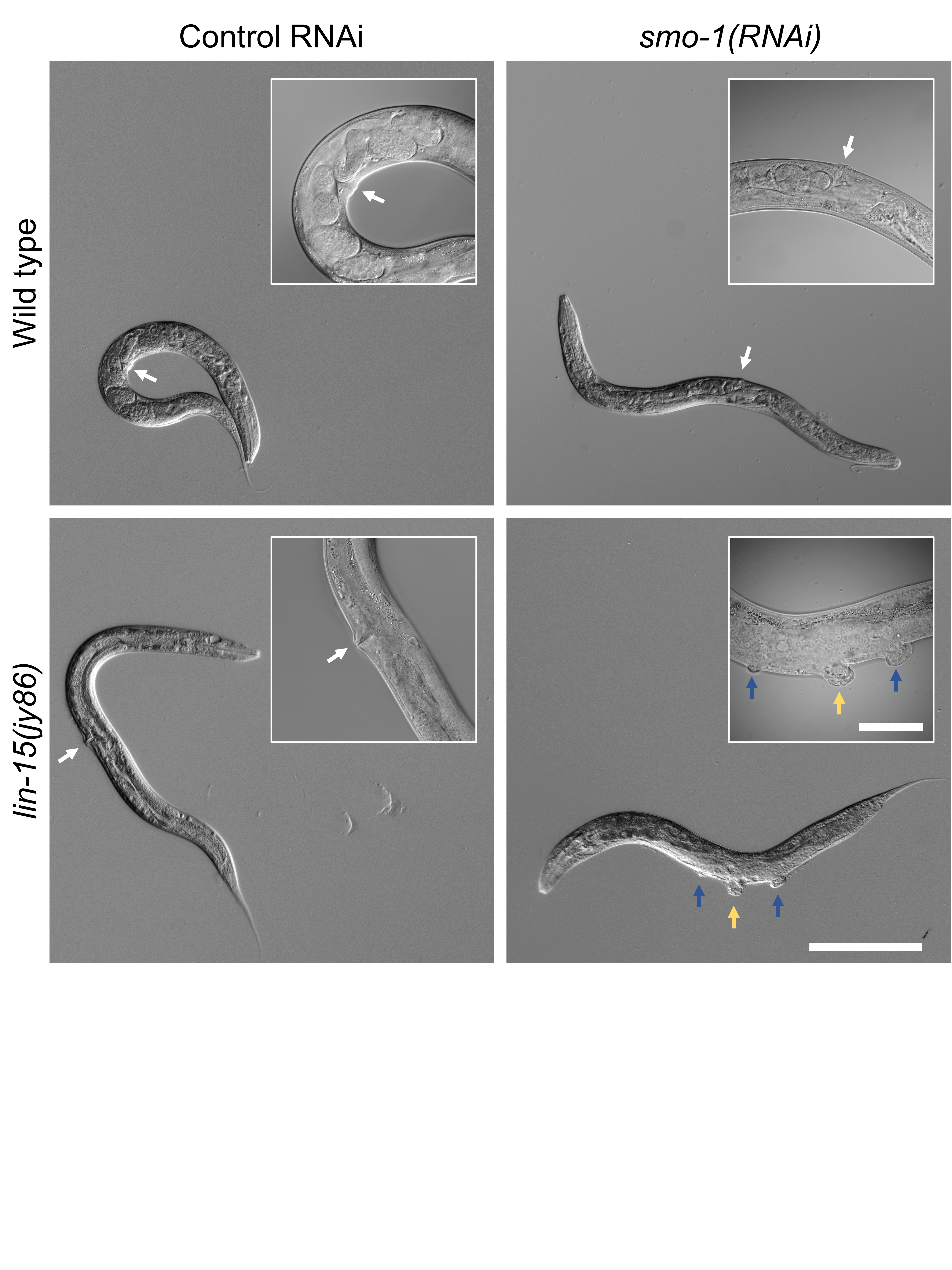

Supplement: S2 Fig — Representative images of developmental defects observed in lin-15B(jy86) mutants after exposure to smo-1 RNAi. Enlarged insets provide a closer view of the vulval region for each corresponding full-body image. White arrows indicate normal vulvas, yellow arrows highlight protruding vulvas with normal localization, and blue arrows mark ectopic protruding vulvas. Scale bars: 200 µm (full-body images), 60 µm (insets). (TIF) [file pgen.1011444.s002.tif]

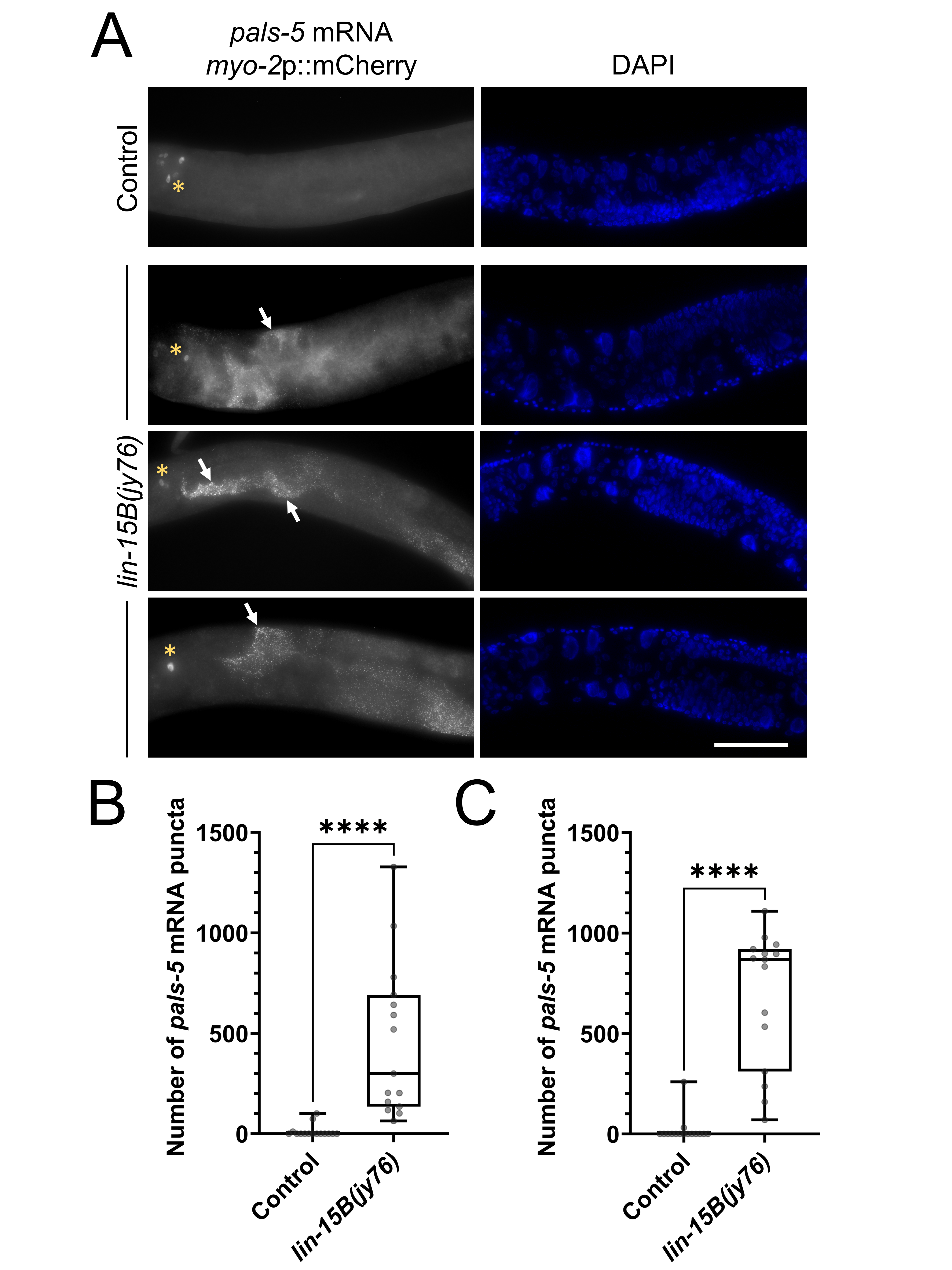

Supplement: S3 Fig — (A) Representative Z-stack maximal projections of smFISH (left) and DAPI staining (right) are shown. White arrows indicate regions with pals-5 mRNA puncta, and yellow asterisks mark the expression of the pharyngeal marker myo-2p::mCherry. Scale bar = 50 µm. (B, C) Quantification of pals-5 mRNA puncta in the first (B) and third (C) intestinal rings, n = 15 animals per sample. In the box-and-whisker plots, the line inside the box represents the median, the box bounds correspond to the 25th and 75th percentiles, and the whiskers extend from the box to the minimum and maximum values. p-values were calculated using a Mann-Whitney test; **** p < 0.0001. (TIF) [file pgen.1011444.s003.tif]

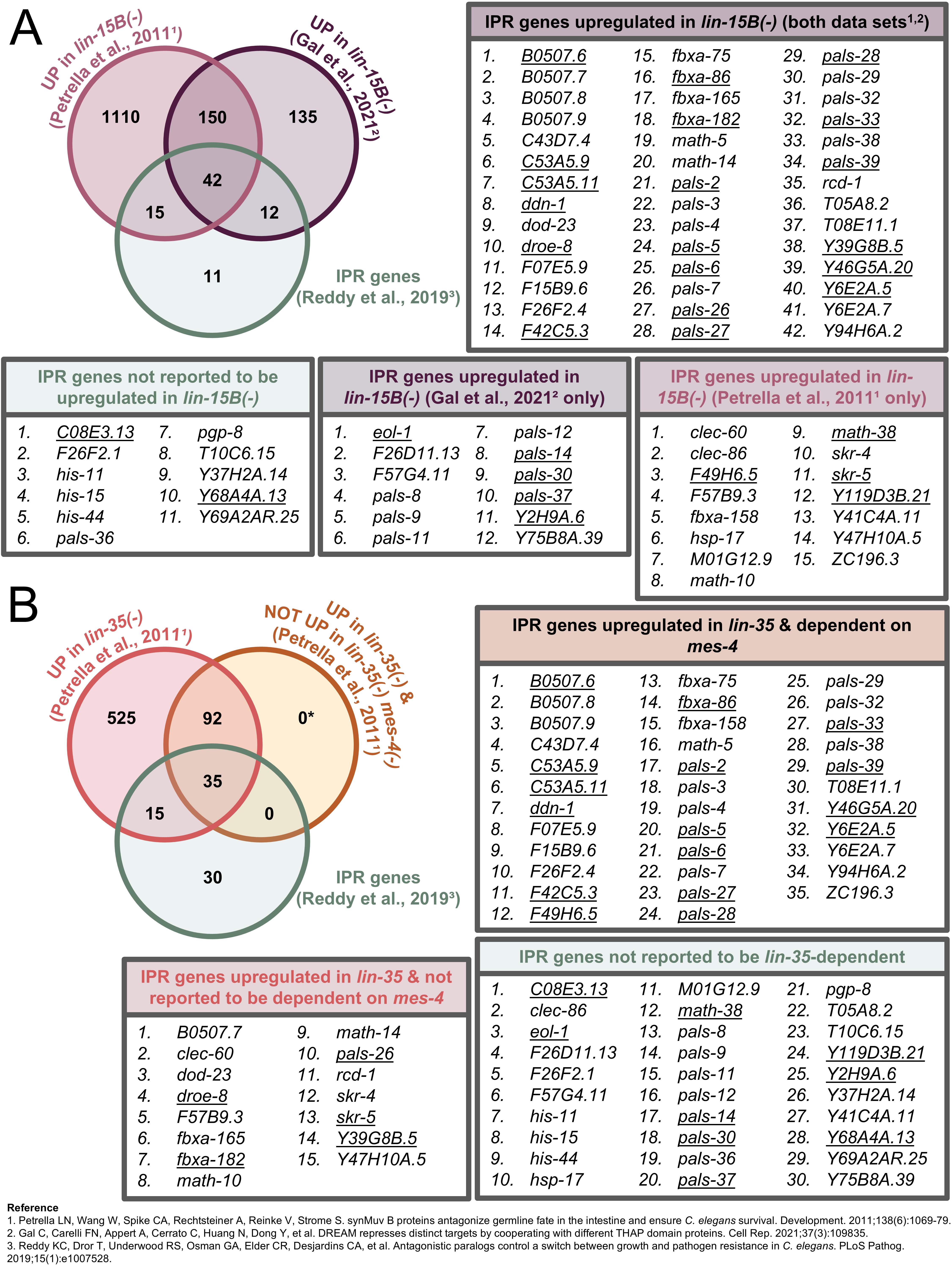

Supplement: S4 Fig — (A, B) In silico analysis of two previously published transcriptomic datasets for lin-15B (A) and lin-35 mutants (B). IPR gene names that are underlined were previously reported to be ZIP-1-dependent. (B) The asterisk indicates a gene that was removed from the original list because it was not found among the genes upregulated in the lin-35(-) mutant. (TIF) [file pgen.1011444.s004.tif]

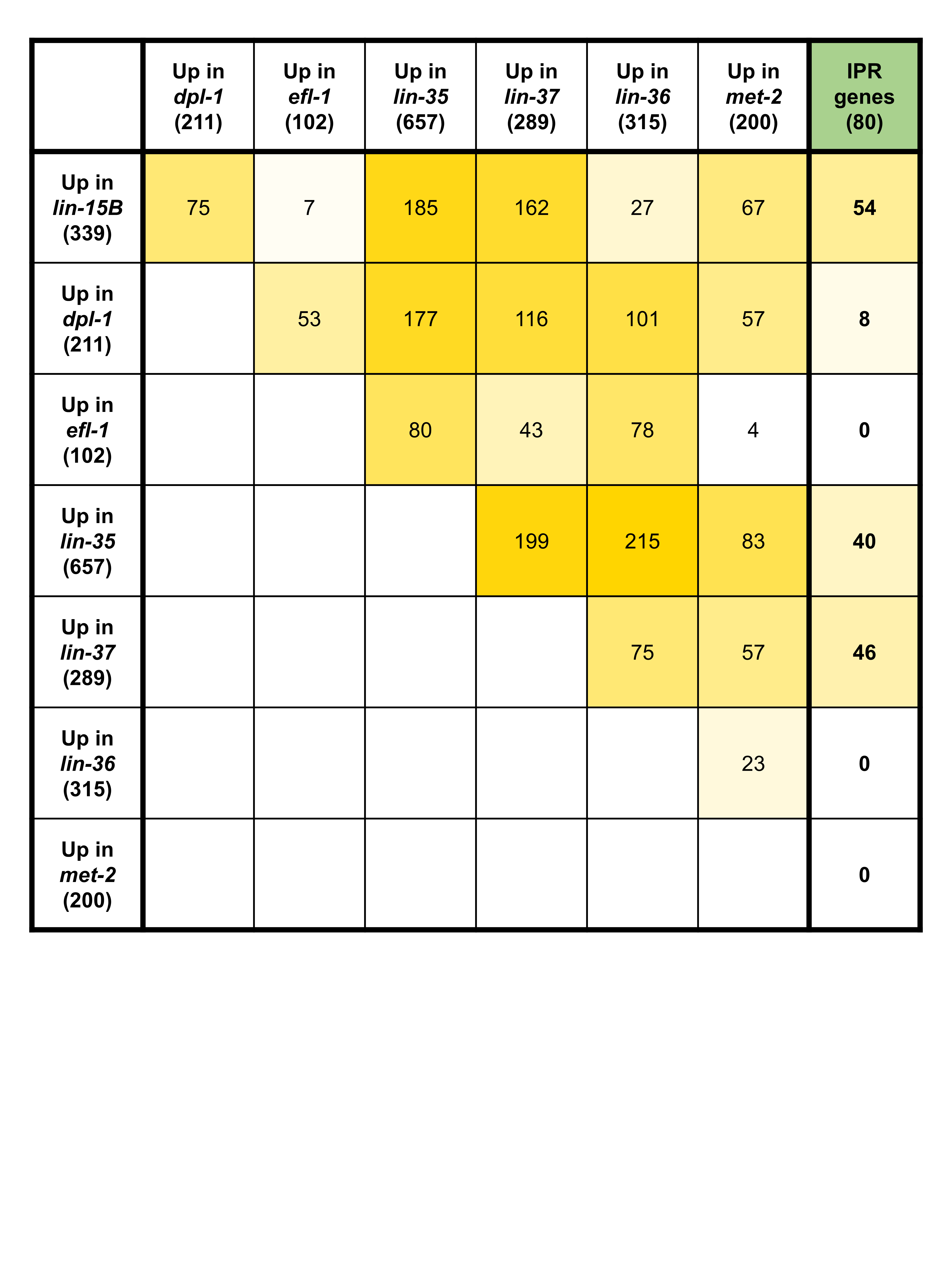

Supplement: S5 Fig — In silico analysis a previously published transcriptomic datasets. The numbers in parentheses indicate the count of upregulated genes in the annotated genetic backgrounds and IPR genes. Numbers in the intersecting cells represent the number of genes common to both datasets. (TIF) [file pgen.1011444.s005.tif]
